# Action Observation With Dual Task for Improving Cognitive Abilities in Parkinson’s Disease: A Pilot Study

**DOI:** 10.3389/fnsys.2019.00007

**Published:** 2019-02-11

**Authors:** Daniele Caligiore, Magda Mustile, Alissa Fineschi, Laura Romano, Fabrizio Piras, Francesca Assogna, Francesco E. Pontieri, Gianfranco Spalletta, Gianluca Baldassarre

**Affiliations:** ^1^Institute of Cognitive Sciences and Technologies, Italian National Research Council, Rome, Italy; ^2^Department of Clinical and Behavioural Neurology, Neuropsychiatry Laboratory, IRCCS, Santa Lucia Foundation, Rome, Italy; ^3^Department of Psychology, University of Stirling, Stirling, United Kingdom; ^4^Department of Neuroscience, Mental Health and Sense Organs, NESMOS, Sapienza University of Rome, Rome, Italy; ^5^Menninger Department of Psychiatry and Behavioral Sciences, Baylor College of Medicine, Houston, TX, United States

**Keywords:** action observation, executive dysfunction, dual task, goal focusing, mirror neurons, Parkinson’s disease

## Abstract

Action observation therapy (AOT) has been recently proposed as a new rehabilitation approach for treatment of motor deficits in Parkinson’s disease. To date, this approach has never been used to deal with cognitive deficits (e.g., deficits in working memory, attention), which are impairments that are increasingly recognized in Parkinsonian patients. Typically, patients affected by these dysfunctions have difficulty filtering out irrelevant information and tend to lose track of the task goal. In this paper, we propose that AOT may also be used to improve cognitive abilities of Parkinsonian patients if it is used within a dual task framework. We articulate our hypothesis by pivoting on recent findings and on preliminary results that were obtained through a pilot study that was designed to test the efficacy of a long-term rehabilitation program that, for the first time, uses AOT within a dual task framework for treating cognitive deficits in patients with Parkinson’s disease. Ten Parkinson’s disease patients underwent a 45-min treatment that consisted in watching a video of an actor performing a daily-life activity and then executing it while performing distractive tasks (AOT with dual task). The treatment was repeated three times per week for a total of 4 weeks. Patients’ cognitive/motor features were evaluated through standard tests four times: 1 month before treatment, the first and the last day of treatment and 1 month after treatment. The results show that this approach may provide relevant improvements in cognitive aspects related to working memory (verbal and visuospatial memory) and attention. We discuss these results by pivoting on literature on action observation and recent literature demonstrating that the dual task method can be used to stimulate cognition and concentration. In particular, we propose that using AOT together with a dual task may train the brain systems supporting executive functions through two mechanisms: (i) stimulation of goal setting within the mirror neuron system through action observation and (ii) working memory and persistent goal maintenance through dual task stimuli.

## Introduction

Although Parkinson’s disease (PD) is mainly considered to be a motor disorder, the importance of non-motor symptoms, such as depression, psychosis and cognitive deficits, has been increasingly recognized (Watson et al., 2010; Pellicano et al., 2017; Schapira et al., 2017). Most of these features have been shown to critically influence patients’ quality of life even in the early stages of the disease (Schrag et al., 2000; Antonini et al., 2012; Pellicano et al., 2015). Cognitive deficits, extensively documented and often defined as *frontal type executive dysfunctions* (Gotham et al., 1988), include impairments of verbal fluency (Obeso et al., 2012), deficits in working memory (Lee et al., 2010), and attention deficits both in the early and moderate stages of the disease (Zhou et al., 2012). These deficits remain difficult to manage with current pharmacological medications, which are mainly directed at addressing motor dysfunctions. While levodopa treatment might only partially restore some cognitive functionalities (Lange et al., 1992; Burn, 2015), a number of these are not affected by dopamine-related treatments and other neuromodulators such as acetylcholine, noradrenaline, and serotonin, might be involved (Biundo et al., 2016; Caligiore et al., 2016; Yang et al., 2016).

Cognitive dysfunctions in PD have also been treated through non-pharmacologic therapies. These approaches are mainly based on cognitive rehabilitation and physical therapy (Murray et al., 2014; Dancis and Cotter, 2015). Cognitive rehabilitation includes four domains of practice (Sinforiani et al., 2004; Biundo et al., 2017): (i) educate the patient about their cognitive weaknesses and strengths; (ii) help to develop lost cognitive skills through retraining; (iii) develop compensatory strategies; and (iv) use the three skills developed through (i–iii) to enhance function in life activities. In PD patients this technique has shown significant, albeit modest, improvement in cognitive domains (París et al., 2011; Cerasa et al., 2014). Physical exercise, which is typically used to address some motor issues of PD (e.g., bradykinesia, postural balance problems) (Tillerson et al., 2003; Ridgel et al., 2009; Allen et al., 2011) has also been identified as a possible treatment for cognitive deficits (Petzinger et al., 2013; Pellicano et al., 2015). Clinical studies have shown that various types of exercise, including aerobic, resistance and dance can improve cognitive functions, learning and memory in PD patients, although the optimal type, amount, mechanisms, and duration of exercise are unclear (Murray et al., 2014). It has also been shown that a combined action of physical therapy and transcranial direct current stimulation may lead to an improvement in cognitive functions, that is, frontal abilities and/or global cognitive ability scales (Manenti et al., 2016). Motor imagery and virtual reality are also two therapeutic approaches that make use of cognitive function to enhance movement and cognitive aspects of people with PD (Mirelman et al., 2013a). For example, it has been shown that an intervention that combines treadmill training augmented by virtual reality reduces fall risk, improves mobility and enhances cognitive function in a diverse group of older adults (Mirelman et al., 2013b).

Recently, the efficacy of two new non-pharmacological PD rehabilitation approaches has been explored: action observation therapy (AOT) and dual-task rehabilitative training. AOT is based on the evidence showing that during observation of a movement, the related action representation “resonates” (i.e., it is reactivated) in our motor system (Rizzolatti et al., 2001). This motor resonance can drive the process of understanding the intention (goal) of the agent performing the action through a facilitatory effect on the motor pathways (Buccino et al., 2001; Wheaton et al., 2004). These processes can drive the learning and acquisition of motor skills in analogous ways as physical exercise (Porro et al., 2007; van der Helden et al., 2010; Higuchi et al., 2012). During AOT, participants are typically required to carefully observe videos showing actions that they later have to execute. It has been shown that AOT can lead to improvements in the performance of movements in PD patients involved in single session experiments (Abbruzzese et al., 2015; Caligiore et al., 2017). In addition, two studies have shown that AOT-based long-term rehabilitation programs, involving repeated sessions spanning over weeks/months, could provide some benefits for PD patients motor recovery (Pelosin et al., 2010; Buccino et al., 2011). To date, AOT has never been used to deal with cognitive deficits in PD.

Dual task requires participants to perform two tasks simultaneously, which interfere with each other, such as engaging in a cognitive task while executing a motor task (Pashler, 1994; Woollacott and Shumway-Cook, 2002). PD patients commonly have difficulty in performing dual task (Benecke et al., 1986; Castiello, 1997). These difficulties can be observed in the performance of two motor tasks, or two cognitive tasks, or combined cognitive and motor tasks (Brown and Marsden, 1991; Woollacott and Shumway-Cook, 2002; Kelly et al., 2012; Nocera et al., 2013). The neural causes of this problem have been related to limited attentional resources, defective central executive function, and less automaticity (Wu and Hallett, 2008; Wu et al., 2014). Dual-task rehabilitative training in PD patients uses the interference between cognitive and motor tasks to obtain functional improvements. Typically, the concurrent performance of motor and cognitive tasks in dual task positively affects the performance of one of them. For example, it may improve gait velocity, stride length, balance or some aspects of cognition (usually mental flexibility and processing speed) (Woollacott and Shumway-Cook, 2002; Silsupadol et al., 2006; Fritz et al., 2015).

In this article, we propose that AOT, traditionally used to improve motor skills in Parkinsonian patients, may also be used to improve their cognitive abilities if it is used within a dual task framework. We articulate our hypothesis by pivoting on recent literature and on preliminary results we obtained through a pilot study we designed to test the efficacy of a long-term rehabilitation program for therapy that, for the first time, uses AOT within a dual task framework to address cognitive impairments in PD. The results of the experiment show that using AOT within a dual task framework leads to relevant improvements in cognitive aspects related to working memory (verbal and visuospatial memory) and attention. In contrast, no significant improvement in motor behavior was found, which is a typical result when using AOT in isolation (Buccino, 2014; Abbruzzese et al., 2015; Caligiore et al., 2017). We propose that AOT with dual task may help patients to deal with the difficulty of filtering out irrelevant information and with the tendency that they have of losing the task goal, which are both features that characterize cognitive deficits in PD (Lee et al., 2010). In particular, we hypothesized that using AOT with a dual task may engage and train goal-centered executive functions (Diamond, 2013) through two interacting mechanisms. First, AOT stimulates goal activation based on motor resonance mechanisms activated within the mirror neuron system (Rizzolatti and Craighero, 2004; Craighero et al., 2007; Thill et al., 2013). Second, during AOT the patient also performs a mathematical or lexical task. The accomplishment of this second goal tends to engage working memory and attention systems that are also involved in the motor tasks (Silsupadol et al., 2006). If the tasks are sufficiently motivating, the goals might be persistently maintained active and guide the performance of suitable actions to accomplish them, notwithstanding the possible cross interference and distraction between them. This exercise results in a cognitive effort that leads to the strengthening of the executive functions.

The executive processes related to goal activation and maintenance are particularly important for PD cognitive dysfunctions and their treatment because they strongly involve the regulation of the brain mechanisms underlying goal-directed behavior by the dopamine and basal-ganglia systems (Balleine and O’Doherty, 2010; Mannella et al., 2016). Indeed, both empirical evidence and computational models show the importance of dopamine and nucleus accumbens for the activation of goals and their persistent permanence in time (Redgrave et al., 2010; Fiore et al., 2014; Floresco, 2015). Important for therapy effectiveness, these processes can also be enhanced by the release of dopamine caused by the engaging and novel features of the tasks used for training (Lisman and Grace, 2005; Mannella et al., 2013).

## Methods

### Patients

A total of ten patients (four women and six men; mean age ±*SD* = 63.9 ± 10.7 years; see Table 1 for more details) with idiopathic PD, according to the United Kingdom Parkinson’s Disease Brain Bank criteria, participated in this study. Inclusion criteria were: moderate Parkinsonian symptoms (1–3 of the Modified Hoehn and Yahr Scale); Mini-Mental State Examination score >24; stable medication regimen; absence of neurological and psychiatric comorbidity; absence of unpredictable motor fluctuations; absence of mood depression according to DSM IV criteria. All participants were right handed as measured by the Edinburgh Handedness Inventory Questionnaire. All procedures were approved by the local ethics committee and written informed consent was obtained from the participants before taking part in the study.

**Table 1:** Data on the PD patients involved in the experiments.

	Mean	*SD*	*SE*	Min	Max
Age (years)	63.9	10.734	3.394	48	77
Education (years)	12.9	4.999	1.581	5	19
Disease duration (years)	5.4	9.931	3.140	42	74
Disease stage (Hoehn and Yahr)	1.55	0.643	0.203	1	2.5
MMSE (Mini Mental State Examination)	28.78	1.334	0.445	26.40	30

### Experimental Procedure

All patients were evaluated four times: 1 month before the onset of treatment (baseline), on the first day of treatment (pre-test), on the last day of treatment (post-test) and 1 month after treatment (follow-up).” The comparison between baseline and pre-test conditions it has been made to ensure that there were no in place strong changes or fluctuations in the cognitive and motor performance. Disease severity was determined by means of the Hoehn and Yahr Scale. The evaluation consisted in a battery of standard tests now illustrated in detail (see Table 2).

**Table 2:** The test battery and the cognitive/motor features tested by it.

Test	Cognitive/motor feature	Test explanation
Rey Auditory Verbal Learning Test	Short and long term auditory-verbal memory, learning strategies, retroactive, and proactive interference, presence of confabulation	Recalling/repetition of 15 unrelated words, repeated over 6 different trials: 5 short term trials and the last after 15 min
Rey-Osterrieth Complex Figure Test/Taylor Figure Test	Perceptual organization, visuospatial ability, visuospatial short and long term memory	Reproduction of a complicated line drawing, first by copying it freehand (recognition), then, after 15 min, drawing it from memory (recall)
Phonological and Semantic Fluency Test	Lexical access and organization	Production of as many words as possible from semantic and phonemic category in a given time
Stroop Color and Word Test	Attention, denomination and cognitive interference	Read as fast as possible 3 different tables, 2 tables in congruous condition and the third in color-word condition
Trail Making Test	Attention, executive functioning, visual scanning, psychomotor abilities	Connect a set of 25 dots (numerical and verbal dots) as quickly and accurately as possible
Wisconsin Card Sorting Test/Weigl Test	Attention shifting, executive functioning, working memory, error monitoring, inhibition of automatic responses, perseveration	Sort the cards based on color, form, or number, without knowing which of the 3 criteria to use/Sort 12 stimulus in different combinations of colors, shapes, symbols, dimensions and thickness, with a sorting strategy
Corsi Block Tapping Test	Spatial attention, visuospatial working memory	Repeat the tapping sequences on a group of 9 identical blocks
Forward/Backward Digit Span Task	Short term verbal memory	Repeat a series of digits forward and backward. The length of the series increases with each correct answer
IADL (Instrumental Activity of Daily Living) and ADL (Activity of Daily Living)	Ability/autonomy in daily living activities	Self evaluation of independence in carrying out 6 basic activities (ADL) and 7 instrumental activities (IADL) necessary for daily self-care
EuroQol rating scale	Self-reported functionality/activity of daily life	Questionnaire with 5 items and 3 levels of evaluation for each item
UPDRS (Unified Parkinson’s Disease Rating Scale) part II	Self-evaluation of motor experiences in daily living activities	Questionnaire with 13 items and 5 levels of evaluation for each item
Jebsen Hand Function Test	Speed of fine and gross hand movements with common objects	Simulation of daily living activities with dominant and non-dominant hand
UPDRS (Unified Parkinson’s Disease Rating Scale) part III	Motor evaluation	Questionnaire with 32 items and 5 levels of evaluation for each item
Tinetti Balance and Gait Assessment Scale	Gait and balance evaluation to predict the risk of falling	The test consists of 2 subscales. Balance: 9 items with a score ranging from 0 to a maximum of 16 Gait: 7 items with a score between 0 and 12

### Neuropsychological Battery

The neuropsychological battery consisted in the evaluation of several cognitive domains. This extensive battery of tests has been used with the aim to test which cognitive domain could be affected or not by the proposed treatment. The following tests were used for this purpose: Rey Auditory Verbal Learning Test (Rey, 1964), Rey-Osterrieth Figure Test (Osterrieth, 1944), Phonological and Semantic Fluency Test (Harrison et al., 2000), Stroop Color and Word Test (Stroop, 1935), Trail Making Test (Reitan, 1958), Wisconsin Card Sorting Test (Grant and Berg, 1948), Corsi Block Tapping Test (Milner, 1971), and the Forward/Backward Digit Span Task (Richardson, 2007). To exclude a learning effect, we used a different version of the Rey Auditory Learning Verbal Test, the Taylor Figure and the Weigl Test as alternative versions of the Rey Figure and Wisconsin Card Sorting Test in the different temporal evaluations (Hawkins et al., 2004).

### Movement/Autonomy in Daily Living Scales

To evaluate movement impairment and autonomy in daily life, the following tests were used: the Unified Parkinson’s Disease Rating Scale (UPDRS– Part II and III) (Ramaker et al., 2002), Tinetti Balance Assessment Scale (Tinetti, 1986; Tinetti et al., 1986), IADL and ADL (Katz, 1963; Lawton and Brody, 1969), EuroQol rating scale (EuroQol Group, 1990).

### Movement Measurements

To evaluate movement fluidity and velocity we used the Jebsen Hand Function Test (Mak et al., 2015). The test was constructed by following the instructions on the authors’ web site^1^ and tested on a matching control group of ten healthy volunteers. The test evaluates the speed of manual movement of daily-living activities through seven subtests performed first with the non-dominant hand and then with the dominant hand. The subtests are: writing a list of 24-letters, reading sentences of 3rd grade difficulty; card turning; picking up small common objects and placing them in a container; stacking checkers; simulated feeding; moving light objects (e.g., empty cans); moving heavy objects (e.g., 1 lb. weighted cans). The execution time of each task, performed first with the non-dominant hand and then with the dominant hand, was measured with a stopwatch. Although the Jebsen Hand Function Test has important limitations since it does not take into account important parameters of movement, it has been used for its ecological validity since it allows the measurement of the speed of movement using objects from daily life (e.g., spoons, cans).

### Treatment and Stimuli

All patients underwent a 45-min AOT with dual task treatment. The treatment was repeated three times per week for a total of 4 weeks (12 sessions in total per patient). During each session, patients were required to observe two videos (each video showed one action to perform) of an actor performing a daily-living action and then executing it. Each video provided a variety of activities: food-related actions (e.g., preparing coffee, pouring water from a bottle into a glass, simulating eating); actions related to hygiene/personal care (e.g., brushing teeth, combing, shaving); actions related to dressing (e.g., putting on shoes or a shirt); general actions (e.g., putting on glasses or a plaster, locking a door). We recorded 40 videos of these actions, with both male and female versions, to avoid gender-related effects. An *ad hoc* questionnaire was then written to pre-test such stimuli. This questionnaire was administered to a sample of 20 healthy subjects who, after watching the video, evaluated each movement on a scale of one to five (one = very little and five = very much) indicating for each movement the degree of difficulty: complex, difficult to perform, familiar, tiring, and typical of everyday life. Based on the results of this questionnaire, we excluded the most complex, difficult, least familiar, most tiring and least typical movements of everyday life. We selected 24 total videos as treatment stimuli, whose order of presentation was exactly the same for each participant. The order of presentation was driven by the complexity and difficulty scores so that the treatment started with the simpler movements and gradually moved to the more complex ones. In each treatment session, patients watched two videos of 6–7 min, each repeated twice. After watching the video, the patients were asked to perform the same action with the same object, several times, for 10 to 15 min. During AOT, the participants were asked to verbalize the actions while they were executing them (for the first 5–7 min). In particular, each patient was asked to explain what s/he was doing at that moment (e.g., ‘I am moving my left hand toward the object’; ‘I am grasping the object with my index and thumb’; ‘I am moving the object toward my mouth’). This verbalization facilitated the maintenance of the focus of attention on the current goal and action performance.

When the patient was able to repeat the action in a fluid and correct manner (i.e., the movement execution was complete and correct for at least three times consecutively), a combination of two different kinds of distracting tasks was proposed (for the remaining 5–7 min) (O’Shea et al., 2002; Brauer and Morris, 2010). These tasks consisted of (a) simple operations of counting backward; (b) simple mathematical operations (e.g., multiply, subtract, divide, add); (c) phonological-lexical tasks: listing alphabetic letters and spelling own name and common words forward and backward; and (d) telling about an episode of own life. These tasks were introduced from the onset of the treatment. Their order was randomized among the subjects. This means that the distracting tasks could be repeated throughout the treatment but not the same day.

### Data Analysis

Data analyses were performed using StatView^TM^ for Windows. For each variable measured, a repeated-measures analysis of variance (ANOVA) was conducted using the conditions “baseline,” “pre-test,” “post-test” and “follow-up” as a within subject factor. The scores obtained in the different tests were corrected with relative cut-offs for age and education. In particular, we followed the correction criteria already used in Carlesimo et al. (1996) and (Caffarra et al., 2002a,b) for the Rey Auditory Verbal Learning Test, Fluency Tests, Rey-Osterrieth Complex figure Test, Corsi, Digit Span Task, Weigl Test, Taylor Figure Test, Stroop Color Word Test; in Folstein et al. (1975) for Mini Mental State Examination; in Greve (2001) for the Wisconsin Card Sorting Test. Fisher’s PLSD (protected least significant difference) *post hoc* tests were also applied to compare the means of the results of the tests in six comparisons: baseline/pre-test; baseline/post-test; baseline/follow-up; pre-test/post-test; pre-test/follow-up; post-test/follow-up. A result was considered statistically significant if the *p*-value was less than 0.05.

## Results

### Comparing PD Patients to Healthy Subjects

We used ten healthy adult participants (three women and seven men; mean age ±*SD* = 61.6 ± 6.5 years; mean education (years) ±*SD* = 15.4 ± 2.6) as a control group for the experimental group of PD patients (mean age ±*SD* = 63.9 ± 10.7 years; mean education (years) ±*SD* = 12.9 ± 4.9) for the Jebsen Hand Function Test. Before analyzing the differences in performance of the test, we tested if there were significant differences between the two groups using age and education as variables. The results of unpaired *t*-tests for these variables show that in both cases there is no statistically significant difference between experimental and control groups (variable: age - Mean difference = -4.400; *t*-value = -0.995; *p*-value = 0.333; variable: education – Mean difference = -2.500; *t*-value = -1.399; *p*-value = 0.178).

### Movement Measurements: Jebsen Hand Function Test

We compared the subtest results for the Jebsen Hand Function Test for the control and experimental groups at baseline. These results represent the average of the execution times (measured with a stopwatch with a resolution of 0.01 s) at different subtests performed with both the dominant and the non-dominant hands. The results of the different *t*-tests show that there is a significant difference in “moving cards,” “moving small objects” (unpaired *t*-test for moving cards: Mean difference = -2.228; *t*-value = -2.900; *p*-value = 0.010; unpaired *t*-test for moving small objects: Mean difference = -2.181; *t*-value = -2.158; *p*-value = 0.045). In addition, the *t*-tests show that the two groups differ significantly in “simulated feeding” subtests when asked to use the non-dominant hand and when asked to use the dominant hand (unpaired *t*-test for simulated feeding – non-dominant hand: Mean difference = -3.978; *t*-value = -2.659; *p*-value = 0.016; unpaired *t*-test for simulated feeding – dominant hand: Mean difference = -4.011; *t*-value = -3.113; *p*-value = 0.006).

### AOT With Dual Task

The results of the statistical analysis (Tables 3, 4) of the test battery show statistically significant differences between different time points of the evaluation (baseline vs. post-test/follow-up; pre-test vs. post-test/follow-up) in the Rey Auditory Verbal Learning Test for both short-term memory test trials (*p* = 0.013), and for long-term memory test trials (*p* = 0.034), in the Stroop Color and Word Tests both in the reading time task (*p* = 0.039) and in the interference task (*p* = 0.043), and in the Rey-Osterrieth Complex Figure Test (*p* = 0.001). Table 3 summarizes the results of the *post hoc* analysis. We found a statistically significant improvement between baseline/post-test conditions and between pre-test/post-test conditions in the Rey Auditory Verbal Learning Test for both short-term memory test trials, and for long-term memory test trials. The *post hoc* analysis also shows a significant improvement between baseline/follow-up and pre-test/follow-up conditions in the reading time during the performance of the Stroop Color and Word Test. Similarly, there is a statistically significant improvement between baseline/post-test conditions in the Stroop Color and Word Test in the Interference Task. For the Rey Auditory Verbal Learning Test, Stroop Color and Word Tests, and Rey-Osterrieth Complex Figure Test there are no significant differences between post-test and follow-up conditions. Finally, the results of the *post hoc* analysis show a significant improvement between baseline/follow-up conditions in long-term visuospatial memory as measured by the Rey-Osterrieth Complex Figure Test.

**Table 3:** The statistically significant results of the *post hoc* tests carried out.

Test	Cognitive function probed	Baseline (mean ±*SD*)	Pre-test (mean ±*SD*)	Post-test (mean ±*SD*)	Follow-up (mean ±*SD*)	Significant comparison	Mean difference	*P*-value
Rey Auditory Verbal Learning Test, STM	Verbal short-term memory	51.3 ± 8.7	50.5 ± 12.5	53.3 ± 8.7	52.5 ± 13.4	Baseline/post-test Pre-test/post-test	–8.786 –7.714	*P*-value = 0.002 *P*-value = 0.007
Rey Auditory Verbal Learning Test, LTM	Verbal long-term memory	11.1 ± 2.2	11.2 ± 3.3	12.5 ± 2.6	11.6 ± 3.4	Baseline/post-test Pre-test/post-test	–2.143 –1.857	*P*-value = 0.008 *P*-value = 0.019
Stroop Color and Word Test	Reading (time)	14.6 ± 4.4	14.0 ± 2.5	13.5 ± 2.4	12.5 ± 2.4	Baseline/follow-up Pre-test/follow-up	+2.794 +1.987	*P*-value = 0.006 *P*-value = 0.042
Stroop Color and Word Test	Cognitive interference (time)	32.1 ± 6.7	33.4 ± 10.2	30.5 ± 7.1	29.7 ± 9.8	Baseline/post-test	+3.263	*P*-value = 0.008
Rey-Osterrieth Complex Figure Test	Perceptual organization and visuospatial long-term memory	19.8 ± 6.3	24.6 ± 5.1	22.9 ± 5.7	25.8 ± 6.5	Baseline/follow-up	–6.857	*P*-value = 0.0004

**Table 4:** Not significant tests.

Test	Cognitive function probed	Baseline (mean ±*SD*)	Pre-test (mean ±*SD*)	Post-test (mean ±*SD*)	Follow-up (mean ±*SD*)
Phonological Fluency Test	Lexical access and organization	47.0 ± 12.3	47.5 ± 14.1	48.8 ± 133	45.1 ± 11.8
Semantic Fluency Test	Lexical access and organization	22.3 ± 6.4	24.2 ± 10.3	26.0 ± 10.2	24.0 ± 9.6
Trail Making Test – part A	Attention, executive functioning, visual scanning, psychomotor abilities	72.3 ± 41.7	54.2 ± 14.1	52,.6 ± 13.4	46.6 ± 9.4
Trail Making Test – part B	Attention, executive functioning, visual scanning, psychomotor abilities	88.4 ± 51.1	89.6 ± 45.1	81.0 ± 51.6	70.9 ± 48.5
Wisconsin Card Sorting Test/Weigl Test	Attention shifting, executive functioning, working memory, error monitoring, inhibition of automatic responses, perseveration	5.2 ± 1.4	3.1 ± 1.0	5.7 ± 0.8	3.4 ± 0.7
Corsi Block Tapping Test	Spatial attention, visuospatial working memory	3.4 ± 1.2	2.9 ± 1.4	2.4 ± 1.6	2.5 ± 1.4
Forward Digit Span task	Short term verbal memory	6.0 ± 0.7	5.9 ± 1.4	5.8 ± 1.4	5.6 ± 0.9
Backward Digit Span Task	Short term verbal memory	4.9 ± 1.2	5.1 ± 0.9	4.6 ± 1.4	4.8 ± 0.7
IADL/ADL	Ability/autonomy in daily living activities	5.5 ± 0.5	5.5 ± 0.5	5,6 ± 0.4	6.0 ± 0.3
EuroQol rating scale	Self-reported functionality/activity of daily life	6.9 ± 0.8	6.8 ± 1.3	6.8 ± 1.2	6.8 ± 1.5
UPDRS (Unified Parkinson’s Disease Rating Scale) part II	Self-evaluation of motor experiences in daily living activities	5.7 ± 4.5	5.2 ± 4.3	4.7 ± 3.4	5.3 ± 4.5
Jebsen Hand Function Test Dominant Hand	Speed of fine and gross hand movements with common objects	52.1 ± 3.8	52.6 ± 7.4	49.5 ± 5.9	54.0 ± 3.9
Jebsen Hand Function Test Non-dominant Hand	Speed of fine and gross hand movements with common objects	84.5 ± 5.4	83.9 ± 6.5	80.3 ± 6.4	84.5 ± 6.7
UPDRS (Unified Parkinson’s Disease Rating Scale) part III	Motor evaluation	13.2 ± 7.3	11.8 ± 6.7	10.6 ± 4.4	11.6 ± 5.2
Tinetti Balance	Gait and balance evaluation to predict the risk of falling	7.0 ± 2,55	8.4 ± 2.6	8.5 ± 2.8	9.5 ± 3.0
Gait Assessment Scale	Gait and balance evaluation to predict the risk of falling	13.7 ± 3.1	14.4 ± 1.3	14.5 ± 1.3	14.3 ± 1.4

Aside from these statistically significant results, the data shows some trends indicating a strong difference between baseline/follow-up conditions (Mean difference = 2.227; *p*-value = 0.057); pre-test/post-test (Mean difference = 2.263; *p*-value = 0.054) in the Stroop Color and Word in the Interference Task, and between baseline/post-test conditions (Mean difference = 2.227; *p*-value = 0.098) in the reading time during the performance of the Stroop Color and Word Test. Finally, there is a strong trend for the Semantic Fluency Test (*p* = 0.080) with a *post hoc* showing a statistically significant difference between baseline and post-test conditions (Mean difference = -4.571; *p*-value = 0.013).

## Discussion

The progression of the PD tends to cause, on average, a progressive deterioration of motor and cognitive capabilities in patients (Antonini et al., 2012; Caligiore et al., 2016; Schapira et al., 2017). The benefits of therapeutic interventions should hence show an interruption of the deterioration trend and, when possible, an improvement of those capabilities. Such benefits might manifest right after the intervention, and hopefully persist in a later follow-up monitoring. In some other cases the beneficial effects might be detected only in the follow-up tests if they require brain consolidation mechanisms to manifest in behavior (Pelosin et al., 2010).

The results show that after the intervention there are significant improvements in both short-term and long-term verbal memory (Rey Auditory Verbal Learning Test), in long-term visuospatial memory (Rey-Osterrieth Complex Figure Test), and in some attentional/focussing aspects (Stroop Tests). Interestingly, for the Rey Auditory Verbal Learning Test, for both short-term memory test trials, and for the long-term memory test trials there are no significant differences between post-test and follow-up conditions. This indicates a persistence of the effect of the treatment 1 month after it ends.

Overall these data suggest that using AOT with a dual task can be effective in the treatment of PD frontal deficits, in particular, to develop improvement in working memory and attention. In particular, the results support our hypothesis for which the use of AOT with a dual task may foster goal setting and maintenance in the presence of distractors by training the working memory and attention executive systems. The results might rely on two mechanisms. First, AOT supports goal focusing through the mirror neuron system whose functioning involves both cortical (Rizzolatti et al., 1996; Fogassi et al., 2005) and sub-cortical areas (Caligiore et al., 2013; Bonini, 2016; Bruni et al., 2018). AOT is based on a motor resonance mechanism, reproduced by the mirror neurons firing (di Pellegrino et al., 1992; Rizzolatti et al., 1996; Buccino et al., 2001; Mukamel et al., 2010) when the participant observes another agent performing *a goal-directed action*, such as, for example, grasping an object (Rizzolatti and Craighero, 2004). Johnson-Frey et al. (2003) have shown through an fMRI study that the human frontal mirror regions are preferentially activated by the sight of images showing a hand grasping an object compared to a hand touching it. This indicates that mirror neurons tend to encode action goals such as the terminal state resulting from a grasping action (e.g., a certain relationship between the hand and the object). Along the same lines, Fogassi et al. (2005) found that some, but not all, mirror neurons in the parietal cortex of monkeys are selective to ultimate (high-level) goals that a given action contributes to obtain (e.g., “grasp to eat” vs. “grasp to place”). Overall, these data indicate that the mirror system involves the representation of goals at different levels of abstraction (Rizzolatti and Craighero, 2004; Craighero et al., 2007; Thill et al., 2013). Thus AOT, leveraging on the motor resonance mechanism reproduced by the mirror neurons firing, might be a relevant means usable in treatments to activate the participants’ goals.

Second, the additional exercises performed during AOT through the dual-task procedure (e.g., math operations, listing alphabetic letters), challenge the working memory functions. In particular, they train the participants’ capacity for maintaining the activation of two goals, one related to the cognitive task and the other related to the motor task. In this way, the role of cognition and concentration is fostered to the detriment of the performance of motor tasks (Woollacott and Shumway-Cook, 2002; Silsupadol et al., 2006).

This employment of AOT within a dual-task setting might be particularly valuable and effective for the treatment of PD symptoms as the activation and focussed maintenance on specific goals strongly relies on the effective functioning of the basal ganglia and dopaminergic systems (Redgrave et al., 2010; Fiore et al., 2014; Floresco, 2015). Given this involvement of the dopaminergic system, the treatment is also expected to be more effective for therapy if the training involves actions that are engaging and have a high functional value for the participants as this results in a stronger stimulation of the dopaminergic system and ventral basal ganglia (Redgrave and Gurney, 2006; Dagher and Robbins, 2009; Baldassarre et al., 2013). In this respect, most participants of this study informally reported that the training was at the same time challenging but also doable. This balanced level of challenge led them to a high engagement that might have played a relevant role in the positive outcome of the treatment (future work should further investigate these motivational aspects).

These mechanisms may also explain why AOT together with the dual task were able to facilitate cognitive improvement but did not lead to a significant motor improvement, as usually found when AOT is used alone. Indeed, the approach used might have in particular focussed the participants’ training on goal-related cognitive processes rather than on motor ones. This perspective is coherent with influential proposals highlighting how PD patients may be facilitated to operate in a goal-directed control mode (Redgrave et al., 2010) and also with literature suggesting that goal-based exercises may be effective in addressing deficits in PD (Petzinger et al., 2013).

While the results achieved support the idea that using AOT together with a dual task could provide a new way to treat cognitive deficits in Parkinson, more work is needed to design studies that compare the effect of combined AOT with dual tasks in control groups (e.g., performing only one activity, namely AOT, or the dual task, or motor exercise). This, together with the low sample size of PD patients, are the main reasons for which we propose this article as a pilot study. These further investigations could allow to study the role of each single component of the therapy (AOT/dual task/motor exercise). For example, it might be possible that the dual task training in isolation could be enough to improve cognitive measures. Moreover, in light of the absence of outcomes in the motor domains obtained in the current experiment, it might also be possible that the dual task component negated the potential benefit of the AOT component if the performance of such cognitive task was prioritized over the motor activity by the participants [see Kelly et al. (2012), for discussion of dual task prioritization in PD]. Overall, the experiment illustrated here shows that the proposed treatment had clinically relevant effects on cognition, but further experiments are needed to understand the contribution of its different components, or of their interaction, to the beneficial effects obtained.

## Conclusion

Several single session experiments (Abbruzzese et al., 2015; Caligiore et al., 2017) and two studies based on a long-term rehabilitation programs (Pelosin et al., 2010; Buccino et al., 2011) have demonstrated the benefits of AOT in PD motor rehabilitation. This research demonstrates for the first time, through a long-term rehabilitative intervention, that AOT could also lead to the development of cognitive improvement in PD patients if used within a dual task framework. We suggest that this happens because AOT with a dual task trains PD patients to better deal with the difficulty of filtering out irrelevant information as well as with the tendency of losing focus on pursued goals, which are both features that characterize the cognitive deficits of PD (Lee et al., 2010) and strongly depend on the dopamine system (Fiore et al., 2014; Floresco, 2015).

## Author Contributions

DC, MM, FEP, GS, and GB conceived the presented idea and developed the theory. DC, MM, FEP, GS, FA, and GB designed the experiments. MM, LR, AF, and FP performed the experiments. DC, MM, LR, and GB analyzed the data. All authors verified the analytical methods, discussed the results, and gave feedback on the manuscript. DC wrote the manuscript.

## Conflict of Interest Statement

The authors declare that the research was conducted in the absence of any commercial or financial relationships that could be construed as a potential conflict of interest.

## Footnotes

1 http://www.rehabmeasures.org/Lists/RehabMeasures/DispForm.aspx?ID=1025
